# When Eating Intuitively Is Not Always a Positive Response: Using Machine Learning to Better Unravel Eaters Profiles

**DOI:** 10.3390/jcm12165172

**Published:** 2023-08-08

**Authors:** Johana Monthuy-Blanc, Usef Faghihi, Mahan Najafpour Ghazvini Fardshad, Giulia Corno, Sylvain Iceta, Marie-Josée St-Pierre, Stéphane Bouchard

**Affiliations:** 1 Unité de Recherche Loricorps, Centre de Recherche de l’Institut Universitaire en Santé Mentale de Montréal, (CR-IUSMM), 7331, Rue Hochelaga, Montreal, QC H1N 3V2, Canada; 2 Département de Sciences de l’Éducation, Université du Québec à Trois-Rivières, 3351, Boulevard des Forges, Trois-Rivières, QC G8Z 4M3, Canada; 3 Département de Mathématiques et d’Informatique, Université du Québec à Trois-Rivières, 3063, Ringuet, Trois-Rivières, QC G8Z 4M3, Canada; 4 Centre de Recherche de l’Institut Universitaire de Cardiologie et de Pneumologie de Québec, Université Laval, Quebec, QC G1V 0A6, Canada; 5 Département de Psychoéducation et de Psychologie, Université du Québec en Outaouais, 283, Boul Alexandre-Taché, Gatineau, QC J8X 3X7, Canada

**Keywords:** body dissatisfaction, intuitive eating, restraint, bulimia, Causal Reasoning, COVID-19

## Abstract

Background: The aim of the present study was to identify eaters profiles using the latest advantages of Machine Learning approach to cluster analysis. Methods: A total of 317 participants completed an online-based survey including self-reported measures of body image dissatisfaction, bulimia, restraint, and intuitive eating. Analyses were conducted in two steps: (a) identifying an optimal number of clusters, and (b) validating the clustering model of eaters profile using a procedure inspired by the Causal Reasoning approach. Results: This study reveals a 7-cluster model of eaters profiles. The characteristics, needs, and strengths of each eater profile are discussed along with the presentation of a continuum of eaters profiles. Conclusions: This conceptualization of eaters profiles could guide the direction of health education and treatment interventions targeting perceptual and eating dimensions.

## 1. Introduction

### 1.1. Holistic Portrait of Eaters

Although eating is an essential need, the act of eating is an expression of our identity in all areas of our lives (biological, cultural, occupational, relational, perceptual, and sensorial) [1,2]. Behaviors as well as attitudes, beliefs, and motivations related to food and eating have been extensively studied. Among these, restrained eating, emotional eating as well as intuitive eating are some of the most studied topics [3]. Restrained eating refers to deliberately attempt to limit the amount and type of food eaten and is often associated with negative body image (e.g., weight and shape concerns and body dissatisfaction) [4,5]. Restrained eating is typically used as a maladaptive strategy to control one’s weight and shape and it implies the use of rigid cognitive and behavioral strategies to control and avoid overeating [3,6,7,8,9]. Emotional eating involves various forms of excessive eating in response to negative emotions (e.g., anxiety, stress, and irritability) [10,11,12,13]. From a psychosomatic perspective, emotional eaters are unable to discriminate hunger from the physiological states that characterize negative emotions [10]. As a result, emotional eaters tend to (over-)eat when experiencing negative emotions [14,15]. Restrained eating and emotional eating have been associated with eating disorder symptomatology, weight problems, and other negative psychological outcomes, such as anxiety, depression, thoughts and behaviors related to self-harm and suicide, as well as challenges in personal relationships and sexuality [8,15,16,17]. In contrast, intuitive eating is a more recent construct that is considered an adaptative nutritional approach [6,18,19]. It is characterized by refusing both dietary restraint and the categorization of “bad” versus “good” foods, as well as the unconditional permission to eat any food when hungry [20]. In other words, complete intuitive eating referred to the “body–food choice” congruence to achieve eating satisfaction and pleasure [20,21,22,23]. Intuitive eating appears to be a protective factor against dysfunctional eating attitudes and behaviors. It is associated with more positive body image, emotional functioning, increased interoceptive awareness, greater weight stability and food intake regulation [24,25,26,27,28,29,30,31].

Beyond knowledge of eating attitudes and behaviors, research on eaters profiles could shed light on the specific characteristics, challenges, and needs of different groups of individuals. Limited research has been conducted to examine the risk factors associated with future manifestation of threshold, subthreshold, or partial eating disorders. The inclusion of subthreshold and partial eating disorders (i.e., Other Specified Feeding or Eating Disorder, OSFED and Unspecified Feeding and Eating Disorders, UFED) [32] is crucial, given their high prevalence and significant morbidity [33]. Despite substantial evidence to the contrary, residual diagnostic categories are often disregarded as “not really serious”, and consequently may have limited access to treatments [34,35]. In fact, Mustelin et al. [35] reported that only 11% of their OFSED/UFED participants received clinical attention, even though it has been suggested that these individuals may benefit from transdiagnostic CBT-based interventions similarly from threshold eating disorders [33]. Identifying distinct vulnerability groups could improve prevention, education, and intervention efforts by targeting different risk and strength factors for each distinct group. Despite the promising advantages in terms of gaining a better understanding and developing tailored interventions, only a few studies to date have examined and proposed profiles of eaters. Tribole & Resch [20,36] identified four eaters profiles: the “careful eater” who tends to overanalyze food choices; the “inattentive eater” who eats without being mindful of what is eaten and why they are eating; the “professional dieter” who follows diet rules based on food restriction; and the “intuitive eater” who eats based on hunger and satiety signals [36]. More recently, Marquis and colleagues [37] distinguished four eaters profiles in the university student community. The “planet–nutrition–kitchen lover” chooses foods based on nutritional and environmental concerns, and typically cooks their own food. The “utilitarian lonely eater” reports no pleasure in eating. They eat a small variety of foods in order survive. The “body-driven eater” is driven by weight concerns and the relationship between nutrition and exercise. The “mindless eater” enjoys snacking, in a variety of contexts and when hungry or not. Acosta and colleagues [38] identified four obesity phenotypes based on pathophysiological variables (i.e., body composition, resting energy expenditure, satiety, satiation, eating behavior, affect, and physical activity), in order to elucidate the complexity and to guide pharmacotherapeutic interventions. The “hungry brain” phenotype is characterized by excessive caloric intake prior to reaching satiety. The “emotional hunger” reported 2.8 times higher levels of anxiety. They also reported higher levels of depressive symptoms, emotional eating, and lower levels of self-esteem and body image compared to the other phenotypes. The “hungry gut” presented a reduced duration of satiety, quantified by accelerated gastric emptying. The “slow burn” is characterized by lower resting energy expenditure, lower muscle mass, and reduced physical activity compared to participants with the non-slow burn obesity phenotype. Research on eaters profiles is still limited. Furthermore, the COVID-19 pandemic led to disruption in lifestyle habits, including those associated with eating, that may have contributed to the emergence of new eaters profiles.

### 1.2. Eating during the COVID-19 Pandemic

Studies have shown that people living with eating disorders experienced a negative impact of the COVID-19 pandemic crisis on eating disorders-related symptoms (e.g., perceived weight gain, fear of weight gain, an increase in body image disturbances, food restriction, overeating/binge eating, purging, and over-exercising) [39,40,41,42,43,44]. Studies have also reported a recurrence of eating disorders symptoms in former eating disorders patients [40,42,43,45,46]. Indeed, Emmelkamp [47] considered eating disorders as one of the five most important direct unhealthy mental health consequences of the pandemic (along with anxiety, depression, post-traumatic stress disorder, and violence). However, clinical eating disorders may represent only the tip of “the iceberg of dysfunctional eating attitudes and behaviors” that spread through the general population in the context of the COVID-19 pandemic [48,49,50,51,52,53,54,55]. The complex act of eating could have become a source of distress in the pandemic context where fear of weight gain is pervasive and lifestyles have become more sedentary [56,57,58]. The recent body image scientific literature about perceptual disturbances (i.e., negative body image, body image disturbances, low body esteem) and dysfunctional eating attitudes and behaviors, including disordered eating (e.g., restrictive eating, binge eating episodes, overeating, emotional eating) offered an interesting picture of the impact of a 2-year legacy of the COVID-19 pandemic (for a review, see Monthuy-Blanc et al. [59]). Drawing from community and (sub-)clinical samples, studies highlight the potential impact of changes in the way people perceive themselves and interact with others (e.g., the popularity of videoconferencing and the over-use of social networking sites due to social isolation) as well as changes in eating attitudes and behaviors, physical activity, and exercise (Monthuy-Blanc et al. [59]). To the authors’ knowledge, only one study has examined profiles of eaters during the COVID-19 pandemic [29]. Using a classical linear hierarchical clustering approach, three eaters profiles emerged along a continuum from dysfunctional pathological eating to functional intuitive eating within a sample of adult participants [29]. At the functional pole of the continuum, the congruent-driven eater has a positive relationship with their body and appears to be well-connected to their body’s internal cues. At the opposite dysfunctional pole, the incongruent-driven eater engages in emotional and external eating, and they are not connected to their body’s signals of hunger and satiety. This lack of awareness of bodily cues can lead to restrictive eating and binge eating. Somewhere between the two poles, the incongruent–perceptual eater self-reports difficulty in connecting and listening to their bodily signals without engaging in dysfunctional attitudes and eating behaviors. The findings from Monthuy-Blanc et al. [29] emerged from exploratory statistical analyses using traditional cluster-analyses procedures. However, less traditional, but potentially more powerful analytical tools are gaining in popularity, such as Machine Learning, which may allow us to refine their initial findings.

### 1.3. Using Machine Learning to Provide a New Empirical Perspective on Data about Eaters Profiles

Advances in data analyses using Machine Learning (ML) offer exciting potential for analyzing body image disturbances datasets [60]. ML can be more powerful than traditional regression methods to unravel and clarify relationships between interdependent variables such as eating attitudes and behaviors [61,62]. As shown in the literature review from Fardouly et al. [60], there are less than a dozen publications using ML to analyze self-reported data on disordered eating and eating disorders. ML offers robust solutions for investigating complex multivariate relationships using innovative mathematical solutions. Haynos et al. [61] found that ML approaches were superior to regressions in predicting the outcome of eating disorders in longitudinal studies. Unfortunately, the elastic net regularized logistic method they used is computationally intensive and suitable for predicting dichotomous variables rather than continuous variables such as measures of attitudes and behaviors. Ren et al. [62] studied maladaptive eating behavior and emotion regulation characteristics using ML to create profiles in their sample of Chinese participants. Their approach to cluster analysis followed a recursive approach in which the algorithm selects points that maximize the difference between participants until it is no longer possible to improve the predictive accuracy of the model. Once the model was built, they tested its performance and confirmed its accuracy. The results revealed the importance of body image flexibility in improving tolerance to psychological distress and body dissatisfaction [62]. However, their approach is very sensitive to the nature of the data, with small changes in the data having a large impact on the results. Their ML method could be improved by pruning (removing) variables from the algorithm, one at a time, to achieve a stable solution where participants remain in their respective clusters despite changes in the dataset, as suggested by Faghihi et al.’s [63] Causal Reasoning work. Such innovative analytical techniques of an iterative ML approach with pruning of each measure to validate cluster models built with more computationally intensive methods than traditional linear regression-based techniques could provide an opportunity to improve the results from Monthuy-Blanc et al. [29] and refine the clusters found using a more traditional analytical approach.

The objective of the current study is to further optimize the initial 3-cluster model proposed by Monthuy-Blanc et al. [29] with ML tools and Faghihi et al.’s [63] iterative pruning and validation approach. Using this approach, it is possible to further explore vulnerability profiles and related challenges of eating attitudes and behaviors and body image perceptions.

## 2. Materials and Methods

### 2.1. Sample

This study used the same initial sample and inclusion criteria as the study by Monthuy-Blanc et al. [29]. A total of 468 participants gave free and informed consent to participate and to have their data used anonymously. Only participants without missing data on the total score once computed according to recommendations were retained for analyses for variables used to identify clusters, resulting in a final sample of 317 participants. Missing data can lead to a lack of precision in the statistical analysis and result in building a biased ML model, leading to incorrect results if the missing values are not handled properly. Although K-nearest and Naïve Bayes can handle data with missing values, most ML algorithms fail when the dataset contains missing values [64]. The final sample retained for ML analysis consisted of 88.6% female (n = 281) and 10.1% male (n = 32) participants, while 0.9% (n = 3) of participants self-identified as gender-fluid/two-spirit or declined to disclose their gender. One person (0.3%) did not provide an answer to the gender question. Age ranged from 14 to 85 years (M = 36.74; SD = 14.52). The mean BMI was 28.34 kg/m^2^ (SD = 10.89), ranging from 10.65 kg/m^2^ to 68.68 kg/m^2^.

### 2.2. Assessment Measures

The same sociodemographic, body perceptions and eating attitudes and behaviors measures were used as in the Monthuy-Blanc et al.’s [29] study. The Body Dissatisfaction and Bulimia subscales of the French very short version of the Eating Disorder Inventory (EDI-VSV) [65] were used to assess self-reported dissatisfaction with one’s own body image and bulimic attitudes and behaviors, respectively. Answers are based on a Likert scale from 0 (“not at all”) to 10 (“extremely”). In our sample, Cronbach’s alpha was fair at 0.72 [66]. Intuitive eating was assessed using the French Canadian version of the Intuitive Eating Scale-2 (IES-2) [67,68]. This self-report questionnaire consists of four subscales (i.e., unconditional permission to eat, reliance on hunger and satiety cues, eating for physical rather than emotional reasons, and body–food choice congruence) and includes a total of 23 items based on a Likert scale ranging from 1 (“strongly agree”) to 5 (“strongly disagree”). In our sample, Cronbach’s alpha was 0.93, indicating excellent internal consistency. The restraint subscale of the Eating Disorder Examination Questionnaire (EDE-Q) [69,70] was used to assess attitudes and behaviors related to restraint. Items are scored on a 7-point scale and a score of ≥4 has been identified as a clinical cut-off [69]. In the current study, Cronbach’s alpha for the restraint subscale was good at 0.84. In order to interpret the characteristics of the eaters profiles identified in the present study, the average scores of body perceptions and eating attitudes and behaviors measures were interpreted in the discussion by referring to information reported in the validation studies of the aforementioned questionnaires. The normative data for body image perceptions and eating attitudes and behaviors measures are reported hereafter. Eating Disorder Inventory (EDI) very short form subscale for body dissatisfaction (EDI-BD) and bulimia (EDI-B) average scores were compared with those reported in the validation study of the Eating Disorder Inventory very short form in a community and a clinical sample (respectively: EDI-BD non-clinical sample: M = 7.48, SD = 6.38; EDI-BD anorexia nervosa sample: M = 15.55, SD = 3.87; EDI-B non-clinical sample: M = 1.94, SD = 3.05; EDI-B anorexia nervosa sample: M = 4.62, SD = 6.02) [65]. Restraint mean scores were compared to the ones reported in the validation study of the Eating Disorder Examination Questionnaire among a community sample and to the clinical cut-off proposed by the authors (EDE-Q-R community sample: M = 1.25, SD = 1.32; EDE-Q-R clinical cut-off: M ≥ 4.00) [69]. The average scores of four dimensions of intuitive eating—unconditional permission to eat, body–food choice congruence, eating for physical reasons rather than emotional reasons, and reliance on hunger and satiety clues—were compared to the ones reported in the validation study of the Intuitive Eating Scale-2 among a community sample (respectively, IES-UPE: range M = 3.46, SD = 0.76–M = 3.70, SD = 0.80; IES-FCC: range M = 3.29, SD = 0.80–M = 3.48, SD = 0.77; IES-EPR: range M = 3.17, SD = 0.90–M = 3.77, SD = 0.85; IES-HSC: range M = 3.52, SD = 0.70–M = 3.72, SD = 0.71) [68].

### 2.3. Procedure

This study was conducted on the same dataset as the Monthuy-Blanc et al.’s [29] study, so the same procedure and choice of measures were used. This cross-sectional study took place from 29 May to 1 September 2020. In Canada, there was a slight reduction in COVID-19-related regulations during the summer of 2020. However, in August 2020, Canada experienced a second wave of the pandemic, and in early October 2020, most regions entered the maximum alert phase, resulting in the closure of non-essential businesses, travel restrictions, bans on gathering, evening curfews, mandatory remote work, and mandatory online schooling [71]. The online survey was distributed through various platforms, including websites, social media, and community listservs for students and professionals. This study was approved by the Ethical Committee of the University of Quebec in Trois-Rivières (CER-20-266-10.21) and was carried out in accordance with current legislation regarding the protection of personal data (Helsinki Declaration of 1975, as revised in 2018, and the Canadian Tri-Council Policy Statement: Ethical Conduct for Research Involving Humans—TCPS 2 of 2018).

### 2.4. Statistical Analyses

In the current study, a ML approach to cluster analysis was applied. The analyses were performed in two steps: (a) identifying an optimal number of clusters, and (b) validating the clustering model of eaters profiles using a procedure inspired by Faghihi et al.’s [63] Causal Reasoning approach, where the validation of the final solution is based on the behavior of the system in the presence and absence of each variable. The method is consistent with Ren et al. [62] approach of first clustering the dataset using ML, then pruning (removing) each variable one at a time, and reclustering the dataset to log and compare participant movements between clusters. It adds to Ren et al. [62] by testing the stability of the clustering model and by applying both linear and nonlinear mathematical models.

## 3. Results

### 3.1. Identification of the Number of Clusters

To begin the analyses, the 3-cluster model of Monthuy-Blanc et al. [29] was replicated and then improved using Agglomerative and Gaussian Mixture clustering to maximize model quality based on log-likelihood and Silhouette scores. We then combined both the Agglomerative and Gaussian Mixture after dimension reduction and clustering algorithms with Principal Component Analysis clustering (PCA, a method that performs best in linear contexts) and with t-distributed Stochastic Neighbor Embedding clustering (t-SNE, a method that performs well in both linear and nonlinear contexts) [72]. It yielded a 7-cluster model.

To empirically assess the goodness of the clustering technique, Silhouette and Normalized Mutual Information (NMI) scores were compared. While the Silhouette score computes the goodness of the clustering technique, NMI calculates the normalization of the mutual information score between zero and one. As shown in Table 1, the best clustering strategies (with the highest coefficient) were Agglomerative clustering with PCA and TSNE.

### 3.2. Validation of the 7-Cluter Model

The model was validated using correlations between measures assessed using a clustermap from Python’s Seaborn library. A clustermap uses hierarchical clustering algorithms to cluster data by similarity and plots a heatmap matrix.

**Figure 1:**
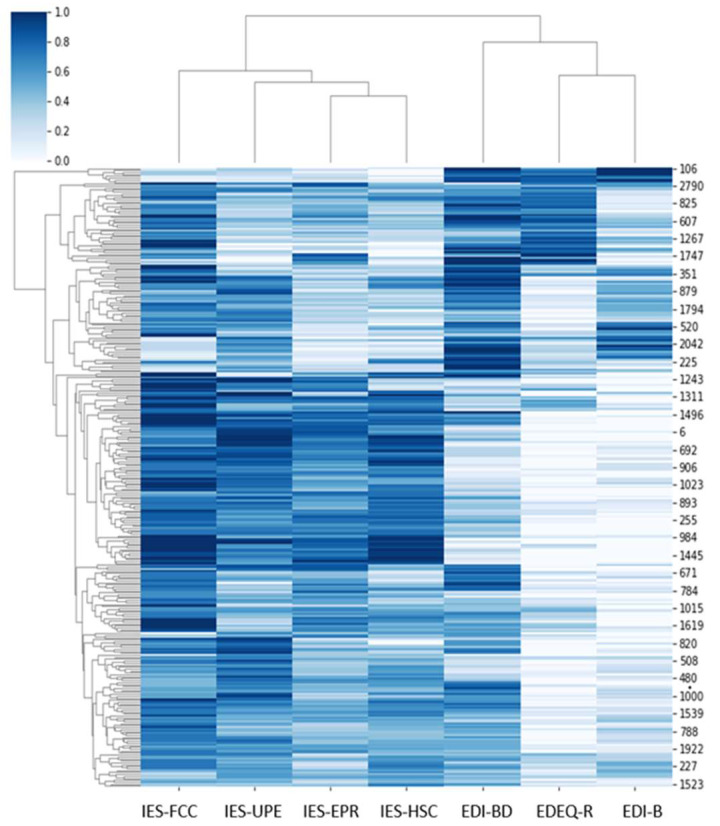
Clustering of participants according to the seven features.

Figure 1 shows the similarity of behavior between variables. For example, the variables “bulimia” (i.e., EDI-B) and “restraint” (i.e., EDE-Q-R) shared more similarities and were more similar to the variable “body dissatisfaction” (i.e., EDI-BD). Figure 1 also shows that the variable “reliance on hunger and satiety cues” (i.e., IES-HSC) shared strong similarities with “eating for physical reasons rather than emotional reasons” (i.e., IES-EPR) and both were similar to “unconditional permission to eat” (i.e., IES-UPE).

To assess the importance of each variable and further validate the number of clusters, the Causal Reasoning approach from Faghihi et al. [63] was adapted to the context of this study and consists of iteratively pruning a variable by reducing its contribution to nearly zero and reclustering the data to compare the number of individuals remaining in the original clusters with the number of individuals changing or creating new clusters. For example, pruning a variable such as “unconditional permission to eat” (i.e., IES-UPE) and reclustering resulted in no change in participants’ assignation to clusters. However, pruning “body dissatisfaction” (i.e., EDI-BD) resulted in the largest changes in clusters.

### 3.3. Descriptive Interpretation of the Clusters

The seven clusters were distinguished from each other by their scores on the various measures of perceptions and eating attitudes and behaviors. Table 2 shows information about the sociodemographic and BMI characteristics of each cluster. Table 3 provides information about the mean scores of each cluster for body dissatisfaction, bulimia, restraint, and intuitive eating. Comparing clusters, Cluster#C7, the “sensitive eater”, showed the lowest dysfunctional perceptions, low dysfunctional attitudes and eating behaviors, and the highest intuitive eating scores (IES-2 average scores between 3.8 and 4.3). In contrast, Cluster#1, the “bulimic eater”, had high dysfunctional perceptions and eating attitudes and behaviors, and the lowest intuitive eating scores (IES-2 average scores between 2.0 and 3.1). Cluster#2, the “restrictive eater”, was characterized by scores similar to Cluster#1 in terms of body dissatisfaction. However, while Cluster#1 had showed higher bulimia scores, Cluster#2 was characterized by higher restraint scores. In addition, the “restrictive eater” (i.e., Cluster#2) was characterized by higher scores on the IES-2 subscale body–food choice congruence compared to the “bulimic eater” (i.e., Cluster#1). Cluster#3, the “partially bulimic eater”, was characterized by lower levels of body dissatisfaction and restraint than Cluster#1 and Cluster#2. Bulimia scores were midway between the two previous clusters. Regarding intuitive eating, the average scores of two subscales (i.e., unconditional permission to eat and reliance on hunger and satiety cues, with average scores between 2.8 and 3.3) were higher than Cluster#1 and Cluster#2’s scores, whereas body–food choice congruence and eating for physical reasons average scores (average scores between 2.9 and 3.7) were halfway between the two previous clusters. Cluster#4, the “body dissatisfied eater”, had the highest mean body dissatisfaction score compared to the other six clusters. It also presented intuitive eating scores similar to the previous three clusters (IES-2 average scores between 3.1 and 3.8), but lower mean scores of bulimia and restraint. Cluster#5, the “almost intuitive eater”, presented lower average levels of body dissatisfaction than Cluster#1, Cluster#2, and Cluster#4, but higher than Cluster#3. However, it had lower average levels of bulimia and restraint and higher mean levels of intuitive eating (mean scores between 3.6 and 4.0) than the previous four clusters. Cluster#6, the “partially restrictive eater”, showed lower average body dissatisfaction and bulimia compared to the previous five clusters, but higher restraint means levels compared to Cluster#3, Cluster#4, and Cluster#5. Regarding intuitive eating, average scores of the subscales were similar to those of the previous clusters with the exception of the body–food choice congruence’s mean score, which was higher than the previous five clusters and was the same as the “sensitive eater” (i.e., Cluster#7).

In order to interpret the clusters intuitively, the average scores of the seven Clusters were compared with the mean scores reported in the validation study of the EDI-VSF [65], the EDE-Q [69], and the IES-2 [68], resulting in a continuum of seven eaters profiles grouped in four categories (see Table 4). Note that comparisons with results from other studies do not refer to statistically significant difference. A detailed discussion of each of the four categories of the continuum is provided in the Discussion.

## 4. Discussion

**Figure 2:**
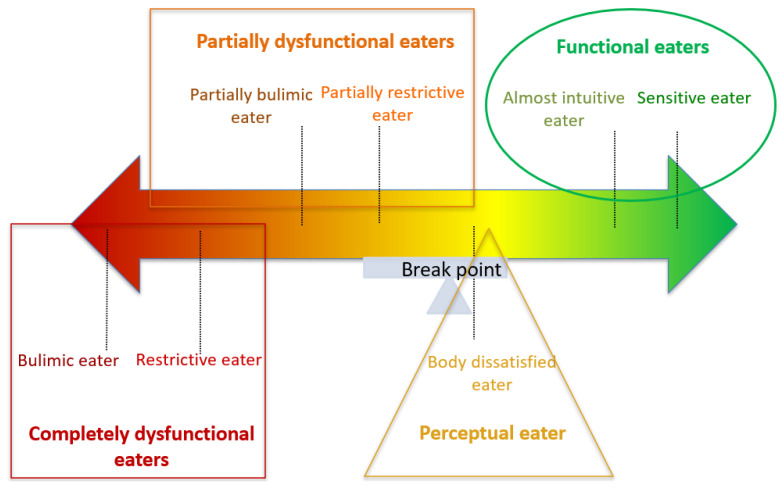
Eaters continuum by perceptual–behavioral eating process based on ML.

Using an approach inspired by Causal Reasoning [63] and adding ML clustering, this study highlights seven distinct eaters profiles in the general population during the COVID-19 pandemic. This reflects the initial continuum of the 3-cluster model proposed by Monthuy-Blanc et al. [29], which distinguishes the congruent-driven eater as a functional eater, the incongruent-driven eater as a dysfunctional eater, and the incongruent–perceptual eater as a critical intersection. The ML analyses allowed us to create a more nuanced and extended continuum of seven eaters profiles (see Figure 2), which were discussed in reference to normative data on body perceptions and eating attitudes and behaviors in clinical and community samples. Profiles with similar characteristics were grouped into four categories: (1) the “completely dysfunctional eaters” (at the dysfunctional pole of the continuum); (2) the “partially dysfunctional eaters”; (3) the “perceptual dysfunctional eater”; and (4) the “functional eaters” (at the functional pole of the continuum). The characteristics of each eater profile are discussed below, along with suggestions for intervention approaches.

### 4.1. Category 1: Completely Dysfunctional Eaters

This category emphasizes the behavioral expression of complete incongruence between body and eating attitudes and behaviors. The two eaters profiles of this category (i.e., Cluster#1 and Cluster#2 eaters) are characterized by perceptual disturbances—in the form of body dissatisfaction—and clinically relevant dysfunctional eating attitudes and behaviors. Furthermore, as the incongruent-driven eaters in the Monthuy-Blanc et al. study [29], these eaters appear to be disconnected from their physical sensations of hunger and satiety, and to eat for reasons other than meeting nutritional requirement.

The eater of Cluster#1, the “bulimic eater”, is characterized by high levels of body dissatisfaction. Compared to the study by Maïano et al. [65], the “bulimic eater” has a level of body dissatisfaction that is above average compared to that reported by their community sample, but below average compared to that reported by their clinical sample (i.e., anorexia nervosa). It is important to note that comparisons with results from other studies do not refer to statistically significant differences; above average/below average are mentioned only to interpret the results in the context of findings from the literature. In terms of dysfunctional eating attitudes and behaviors, Custer#1 has the highest bulimia scores. In fact, when compared to the study by Maïano et al. [65], this eater is characterized by above average levels compared to those reported by both their community and clinical samples. On the other hand, when compared to the study of Fairburn and Cooper’s [73] study, the average levels of restraining are above average compared to those reported by the community sample, but below average compared to their proposed clinical cut-off (i.e., ≥4). The Cluster#1 eater has below average intuitive eating scores compared to those reported by the community sample of Tylka and Kroon Van Diest’s [68] study, on all four intuitive eating dimensions.

Similar to the eater in Cluster#1, the eater in Cluster#2, the “restrictive eater”, presents above average levels of body dissatisfaction compared to those reported by Maïano et al. [65] in their community sample, but below average levels compared to their clinical sample. The “restrictive eater” shows high levels of restraint. In fact, this eater presents above average levels when compared to those presented by Fairburn and Cooper’s [73] study, exceeding the clinical cut-off (i.e., ≥4). In terms of bulimic attitudes and behaviors, this eater has above average levels compared to the community sample, but below average levels compared to those reported by the clinical sample of Maïano et al.’s [65] study. The intuitive eating scores of this eater are similar to those reported by the “bulimic eater” with the exception of the body–food congruence subscale. In fact, the “restrictive eater” has above average levels of body–food congruence than those reported by Tylka and Kroon Van Diest [68]. Moreover, the Cluster#2 eater shows similarities with the professional dieter identified by Tribole and Resch [36], who follows dietary rules based on food restriction.

In summary, the “completely dysfunctional eaters”, present a disconnection between body and eating attitudes and behaviors that, together with high body dissatisfaction, may have led to a self-perpetuating dysfunctional cycle of restraint and bulimic attitudes and behaviors. The “bulimic eater” and the “restrictive eater” already have clinical symptoms related to eating pathology. These two eaters require comprehensive assessment and prompt treatment interventions, as they may meet the diagnostic criteria for eating disorders or subthreshold and partial eating disorders. From an interventional perspective, eaters in this category could benefit from the well-validated Cognitive Behavioral Therapy and Eating Disorders (CBT-E) [74]. This approach provides an account of the “transdiagnostic” theory that overlaps different types of eaters, which may be particularly appropriate for our dimensional approach to eaters profiles.

### 4.2. Category 2: Partially Dysfunctional Eaters

This second category includes two eaters (i.e., Cluster#3 and Cluster#6) who are less dissatisfied with their body, but who still have a problematic relationship between their body perceptions and their eating attitudes and behaviors.

The eater in Cluster#3, the “partially bulimic eater”, presents below average levels of body dissatisfaction compared to the community sample of Maïano et al.’s [65] study, as well as below average levels of restraint compared to the community sample of Fairburn and Beglin’s [69] study. However, this eater still shows above average levels of bulimic attitudes and behaviors compared to the community sample of Maïano et al.’s [65] study. In terms of the intuitive eating dimensions, the Cluster #3 eater presents a profile similar to the Cluster#2 eater.

As the Cluster#3 eater, the Cluster#6 eater, the “partially restrictive eater”, presents below average levels of body dissatisfaction when compared to those reported by the community sample in Maïano et al.’s [65] study. However, unlike the “partially bulimic eater”, the “partially restrictive eater” presents above average levels of restraint and lower levels of bulimic attitudes and behaviors when compared to Fairburn and Beglin’s [69] and Maïano et al.’s [65] studies, respectively. This eater shows below average levels of unconditional permission to eat and reliance on hunger and satiety cues than those reported by the community sample in Tylka and Kroon Van Diest’s [68] study. However, the “partially restrictive eater” has levels of eating for physical reasons similar as those reported by Tylka and Kroon Van Diest [68], and above average levels of body–food congruence.

In summary, the “partially dysfunctional eaters” do not appear to be highly dissatisfied with their body image. However, their profiles indicate a problematic relationship between their body signal and their eating behaviors. In addition, they report dysfunctional eating attitudes and behaviors. From an interventional perspective, this category of eaters, which could be very close to subclinical eating disorders, needs interventions and health education according to an intervention continuum of dysfunctional eating attitudes and behaviors [59]. The goal of these interventions can vary and include both educational efforts to promote healthy eating and an emphasis on intuitive eating to improve food well-being, as well as a focus on self-compassion and self-acceptance to promote food wellness [75]. To target perceptual and bodily cues related to eating behaviors, promising programs focus on perceptual training and eating without worries. Perceptual training, which is reminiscent of yoga (such as intuitive movement), enables the connection between mind–body experience and increases the sense of embodiment [75]. Once perceptions and congruence between bodily signals and eating are restored, an adaptive nutritional approach such as intuitive eating [20] may be appropriate to avoid the negative effects of restrained eating [19,76]. Intuitive movement and eating—as sensory input [77]—are used in recent health and health education interventions for dysfunctional eating attitudes and behaviors [20,75].

### 4.3. Category 3: Perceptual Eater

Category 3 is characterized by one eater profile, the Cluster#4 eater, the “body dissatisfied eater”. This eater experiences the highest levels of body image dissatisfaction but does not engage in dysfunctional eating attitudes and behaviors. In fact, the Cluster#4 eater shows above average levels of body dissatisfaction compared to those reported by Maïano et al. [65] in their community sample, but below average levels when compared to their clinical sample. In addition, Cluster#4 eater shows below average levels of bulimic and restraining attitudes and behaviors when compared to those reported by Maïano et al.’s [65] and Fairburn and Beglin’s [69] studies. Furthermore, the intuitive eating profile of Cluster#4 eater is similar to that of Cluster#6 eater.

In summary, the eater in this category is in a potentially high-risk situation. The high levels of body dissatisfaction are alarming given the well-documented role of body image dissatisfaction in the onset and maintenance of eating disorders and other mental health conditions [16,78,79]. This category may represent the deep part of the “iceberg of dysfunctional eating attitudes and behaviors” [48,49,50,51,52,53,54,55]. They already show high levels of body dissatisfaction and some problems in the relationship between their body and their eating attitudes and behaviors. However, they do not yet actively engage in bulimic or restrained eating attitudes and behaviors. From an intervention perspective, this category corresponds to the “breakpoint” of the eaters’ continuum and may require interventions that target body image as the core of dysfunctional eating attitudes and behaviors. Cognitive behavioral interventions for body image, based on Cash’s cognitive behavioral model, have been extensively studied and shown empirical support for addressing body image concerns in different populations (for a comprehensive review, see Lewis-Smith et al. [80]). These interventions have been validated in a variety of formats, including individual, group-based, online, and self-help book formats. Cognitive behavioral interventions aim to modify irrational and dysfunctional thoughts, emotions, and behaviors [81]. This is accomplished through the use of techniques such as self-monitoring, cognitive restructuring, psychoeducation, desensitization, exposure, and response prevention. Cash’s cognitive behavioral approach to body image interventions has also influenced the development of third-wave cognitive behavioral approaches, such as Dialectical Behavioral Therapy (DBT) and Acceptance and Commitment Therapy (ACT), which have gained popularity over the last decade in addressing body image concerns. However, current evidence suggests that DBT and ACT interventions have demonstrated potential efficacy in addressing eating pathology and body image concerns in specific populations, including DBT for adult women with eating disorder symptoms and ACT for bariatric patients and individuals participating in weight management programs (for a detailed review, see Lewis-Smith et al. [80]).

### 4.4. Category 4: Functional Eaters

The fourth category includes two eaters profiles (i.e., Cluster#5 and Cluster#7 eaters) that could be placed on the functional pole of the eaters continuum. The Cluster#5 eater, the “almost intuitive eater” has below average levels of body dissatisfaction compared to those reported by the community sample in Maïano et al.’s [65] study. In addition, this eater presents below average levels of restraints and bulimia when compared to the ones reported by Maïano et al.’s [65] and Fairburn and Beglin’s [69] studies. The “almost intuitive eater” presents average levels of intuitive eating similar (for the subscales eating for physical reasons and reliance on hunger and satiety clues) or above average (for the subscales unconditional permission to eat and body–food congruence) to those reported in Tylka and Kroon Van Diest [68] study.

The eater in Cluster#7, the “sensitive eater” presents a similar profile to the eater in Cluster#5 in terms of dysfunctional eating (bulimia and restraint) and an even better profile in terms of intuitive eating, and differs in terms of perception (body dissatisfaction). In fact, the “sensitive eater” shows above average levels in all dimensions of intuitive eating compared to those reported by the community sample in Tylka & Kroon Van Diest’s [68] study.

In summary, the “functional eaters” have low levels of body image dissatisfaction and a positive relationship between their body cues and their eating attitudes and behaviors. It is possible that the majority of these subjects are healthy and do not require intervention. However, preventive health education interventions could be implemented to impede eating disorders and partial eating disorders before they ever occur. The recent fourth generation of primary prevention programs on dysfunctional eating attitudes and behaviors advocates the importance of a “blind target” and integrates physical and mental health, simultaneously (targeting both obesity and dysfunctional eating attitudes and behaviors) to avoid conflicting messages from obesity-only and eating disorders-only programs, or the risk of being fascinated by alarmist message about complications of eating disorders [76,82,83,84]. In addition,, these programs should focus on protective factors, such as positive physical self-perceptions, in an ecological environment [83,85,86]. 

### 4.5. Strengths and Limitations

The main strength of this study is the adoption of a novel approach inspired by Causal Reasoning [63]. This study adds to the small number of studies that have taken advantage of the possibilities offered by the ML approach to unravel and clarify the complex relationships between interdependent variables related to eating attitudes and behaviors. Compared to previous works in this field [61,62], the approach used in the present study allows us to implement both linear and nonlinear mathematical models and to obtain results that are more stable and less sensitive to changes in the data [62]. Finally, from a theoretical perspective, the use of ML to better unravel eaters profiles allows us to think differently about mental and physical health. By analyzing a large amount of data with nuances that escape the naked eye, the professional can identify quickly, act early, predict accurately, track daily, and increase accessibility. However, a human is still needed to review the results of the analysis produced by the ML algorithms. Their role is to make sense of these results or to ensure that the data processed by the algorithm is not biased or altered. The current study is not without limitations. First, the cross-sectional design of the study limits our ability to draw conclusions about causal relationships between study variables. Longitudinal studies could provide information about the contribution of specific variables in determining the emergence of dysfunctional eating attitudes and behaviors. Second, because the sample of this study consisted of a majority of self-identified women, the results may not be generalizable to individuals who self-identify with other genders. Future studies should integrate both sex at birth and gender in order to explore and account for biological sex and gender differences and associations. Another potential limitation may be related to the choice of assessment methods. For example, although the EDI-2 is a reliable assessment and discriminating tool for eating disorder psychopathology, its third edition (EDI-3) [87] shows experimental superiority, because it includes a wide range of individuals with different eating disorders, including Binge Eating Disorder and OSFED/UFED [88,89]. In addition, it shows improved reliability compared to the EDI-2 [89]. Finally, future studies should go beyond the analysis of exclusively self-reported variables and also include physiological and anthropometric measures (i.e., body composition, basal metabolic rate, and for individuals under 18 years of age, BMI should be calculated according to the Cole classification system by sex and age group), as well as ecological momentary data, in order to have a holistic representation of the characteristics of eaters profiles [90,91].

## 5. Conclusions

In this study, the contribution of ML has allowed us to refine the understanding of the act of eating by identifying other clusters and sub-clusters with eaters profiles that already exist in the literature. This conceptualization (continuum) of eaters could guide the direction of health education and treatment interventions targeting perceptual and eating dimensions. Because five eaters profiles show a problematic relationship between their body perceptions and their eating attitudes and behaviors, interventions that focus restrictively on intuitive eating should be carefully evaluated taking into account the challenges related to the congruence between body and eating that characterize this approach.

## Figures and Tables

**Table 1:** Comparison of the results from the different clustering techniques used.

	Silhouette	NMI_Score
Agglomerative Clustering with t-SNE	0.175	0.223 *
Gaussian Mixture Clustering with t-SNE	0.173	0.222
Agglomerative Clustering with Original Dataframe	0.197	0.220
Gaussian Mixture Clustering with PCA	0.120	0.218
Agglomerative Clustering with PCA	0.205 *	0.214
Gaussian Mixture Clustering with Original Dataframe	0.042	0.205

Note. NMI_score = Normalized Mutual Information scores; PCA = Principal Component Analysis clustering; t-SNE = t-distributed Stochastic Neighbor Embedding clustering. * = *p* < 0.05.

**Table 2:** Sociodemographic characteristics and BMIs by cluster.

	Cluster#1	Cluster#2	Cluster#3	Cluster#4	Cluster#5	Cluster#6	Cluster#7
	n (%) or M ± SD	n (%) or M ± SD	n (%) or M ± SD	n (%) or M ± SD	n (%) or M ± SD	n (%) or M ± SD	n (%) or M ± SD
N (%)	44 (13.9)	39 (12.34)	49 (15.56)	34 (10.7)	47 (14.8)	30 (9.5)	74 (23.3)
Females	42 (95.5)	36 (92.3)	44 (89.8)	32 (94.1)	42 (89.4)	26 (86.7)	59 (79.7)
Males	2 (4.5)	2 (5.1)	5 (10.2)	2 (5.9)	4 (8.5)	2 (6.7)	15 (20.3)
Other ^a^	/	1 (2.6)	/	/	1 (2.1)	2 (6.6)	/
Age	37.72 ± 14.27	35.68 ± 14.03	32.06 ± 11.48	41.03 ± 14.55	36.74 ± 15.15	37.57 ± 12.64	37.35 ± 16.52
BMI	37.97 ± 17.44	27.36 ± 9.52	26.62 ± 8.42	31.08 ± 8.42	25.68 ± 6.66	26.86 ± 7.23	25.27 ± 9.10

Note. M, mean; SD, standard deviation; BMI, body mass index. ^a^ participants self-identified as gender-fluid/two-spirit or refused to state their gender or did not provide an answer to the gender question.

**Table 3:** Descriptive statistics for eating attitudes and behaviors presented by cluster.

	Cluster#1	Cluster#2	Cluster#3	Cluster#4	Cluster#5	Cluster#6	Cluster#7
	M ± SD	M ± SD	M ± SD	M ± SD	M ± SD	M ± SD	M ± SD
EDI-BD	9.2 ± 1.47	8.4 ± 2.06	5.9 ± 1.61	9.4 ± 1.14	6.1 ± 1.05	5.5 ± 1.17	2.9 ± 0.98
EDI-B	7.0 ± 1.80	2.5 ± 1.63	3.4 ± 1.05	1.1 ± 1.06	0.7 ± 0.71	0.4 ± 0.65	0.8 ± 1.00
EDE-Q-R	1.9 ± 1.69	4.7 ± 0.78	1.1 ± 1.03	0.8 ± 0.83	0.3 ± 0.36	2.0 ± 0.99	0.3 ± 0.50
IES-UPE	3.0 ± 0.88	2.2 ± 0.74	3.3 ± 0.55	3.3 ± 0.96	3.8 ± 0.68	3.0 ± 0.73	4.0 ± 0.67
IES-FCC	3.1 ± 1.06	3.8 ± 0.81	3.7 ± 0.72	3.8 ± 0.83	4.0 ± 0.63	4.3 ± 0.65	4.3 ± 0.63
IES-EPR	2.3 ± 0.55	3.1 ± 0.67	2.9 ± 0.54	3.2 ± 0.73	3.6 ± 0.58	3.6 ± 0.61	3.8 ± 0.60
IES-HSC	2.0 ± 0.72	2.2 ± 0.77	2.8 ± 0.71	3.1 ± 0.9	3.7 ± 0.78	3.0 ± 0.74	3.9 ± 0.82

Note. M, mean; SD, standard deviation; EDI-BD, body dissatisfaction subscale of the eating disorder inventory questionnaire; EDI-B, bulimia subscale of the eating disorder inventory questionnaire; EDE-Q-R, restraint subscale of the rating disorder examination questionnaire; IES-UPE, unconditional permission to eat subscale of the intuitive eating test; IES-FCC, body–food choice congruence subscale of the intuitive eating test; IES-EPR, eating for physical reasons rather than emotional reasons subscale of the intuitive eating test; IES-HSC, reliance on hunger and satiety cues subscale of the intuitive eating test.

**Table 4:** Categories of eaters profiles placed on a continuum.

	Cluster#1	Cluster#2	Cluster#3	Cluster#6	Cluster#4	Cluster#5	Cluster#7
EDI-BD Co: 7.48 (6.38) Cl: 15.55 (3.87)	9.2 (1.47) >Community <Clinical	8.4 (2.06) >Community <Clinical	5.9 (1.61) <Community <Clinical	5.5 (1.17) <Community <Clinical	9.4 (1.14) >Community <Clinical	6.1 (1.05) <Community <Clinical	2.9 (0.98) <Community <Clinical
EDI-B Co: 1.94 (3.05) Cl: 4.62 (6.02)	7.0 (1.80) >Community >Clinical	2.5 (1.63) >Community <Clinical	3.4 (1.05) >Community <Clinical	0.4 (0.65) <Community <Clinical	1.1 (1.06) <Community <Clinical	0.7 (0.71) <Community <Clinical	0.8 (1.00) <Community <Clinical
EDE-Q-R Co: 1.25 (1.32) Cl: ≥4	1.9 (1.69) >Community <Clinical	4.7 (0.78) >Community >Clinical	1.1 (1.03) <Community <Clinical	2.0 (0.99) >Community <Clinical	0.8 (0.83) <Community <Clinical	0.3 (0.36) <Community <Clinical	0.3 (0.50) <Community <Clinical
IES-UPE Co:3.46 (0.76)–3.70 (0.80)	3.0 (0.88) <Community	2.2 (0.74) <Community	3.3 (0.55) <Community	3.0 (0.73) <Community	3.3 (0.96) <Community	3.8 (0.68) >Community	4.0 (0.67) >Community
IES-FCC Co: 3.29 (0.80)–3.48 (0.77)	3.1 (1.06) <Community	3.8 (0.81) >Community	3.7 (0.72) >Community	4.3 (0.65) >Community	3.8 (0.83) >Community	4.0 (0.63) >Community	4.3 (0.63) >Community
IES-EPR Co: 3.17 (0.90)–3.77 (0.85)	2.3 (0.55) <Community	3.1 (0.67) <Community	2.9 (0.54) <Community	3.6 (0.61) =Community	3.2 (0.73) =Community	3.6 (0.58) =Community	3.8 (0.60) =Community
IES-HSC Co: 3.52 (0.70)–3.72 (0.71)	2.0 (0.72) <Community	2.2 (0.77) <Community	2.8 (0.71) <Community	3.0 (0.74) <Community	3.1 (0.9) <Community	3.7 (0.78) =Community	3.9 (0.82) >Community

Note. Co, community sample; Cl, clinical (Eds) sample; <, lower than; >, higher than; EDI-BD, body dissatisfaction subscale of the eating disorder inventory questionnaire; EDI-B, bulimia subscale of the Eating disorder inventory questionnaire; EDE-Q-R, restraint subscale of the eating disorder examination questionnaire; IES-UPE, unconditional permission to eat sub-scale of the intuitive eating test; IES-FCC, body–food choice congruence subscale of the intuitive eating test; IES-EPR, eating for physical reasons rather than emotional reasons subscale of the intuitive eating test; IES-HSC, reliance on hunger and satiety cues subscale of the intuitive eating test. Dark red color, severely dysfunctional; red color, highly dysfunctional; dark yellow color, average; green color, highly functional.

## Data Availability

The data presented in this study are available on request from the corresponding author. The data are not publicly available due to ethical restrictions.
